# Composition of the Cockroach Gut Microbiome in the Presence of Parasitic Nematodes

**DOI:** 10.1264/jsme2.ME16088

**Published:** 2016-08-11

**Authors:** Cláudia S. L. Vicente, Sota Ozawa, Koichi Hasegawa

**Affiliations:** 1 Department of Environmental Biology, College of Bioscience & Biotechnology, Chubu University1200 Matsumoto, Kasugai, Aichi 487–8501Japan; 2 NemaLab/ICAAM—Instituto de Ciências Agrárias e Ambientais Mediterrânicas, Departamento de Biologia, Universidade de ÉvoraNúcleo da Mitra, Ap. 94, 7002–554 ÉvoraPortugal

**Keywords:** cockroach, gut microbiome, multi-trophic interactions, parasitism, nematodes

## Abstract

Cockroaches are parasitized by thelastomatid nematodes, which live in an obligate manner in their hindgut and interact with the resident microbial community. In the present study, a composition analysis was performed on the gut microbiome of *Periplaneta fuliginosa* and *P. americana* to investigate natural and artificial infection by thelastomatid nematodes. Nine libraries of the 16S rRNA gene V3–V4 region were prepared for pyrosequencing. We examined the complete gut microbiome (fore-, mid-, and hindgut) of lab-reared *P. fuliginosa* naturally infected with the parasitic nematode *Leidynema appendiculatum* and those that were nematode-free, and complemented our study by characterizing the hindgut microbial communities of lab-reared *P. americana* naturally infected with *Hammerschmidtiella diesingi* and *Thelastoma bulhoesi*, artificially infected with *L. appendiculatum*, and those that were nematode-free. Our results revealed that the fore- and midgut of naturally infected and nematode-free *P. fuliginosa* have close microbial communities, which is in contrast with hindgut communities; the hindgut communities of both cockroaches exhibit higher microbial diversities in the presence of their natural parasites and marked differences were observed in the abundance of the most representative taxa, namely *Firmicutes*, *Proteobacteria*, and *Bacteroidetes*. Our results have provided basic information and encourage further studies on multitrophic interactions in the cockroach gut as well as the thelastomatid nematodes that play a role in this environment.

The species composition of the insect microbiome is very complex and has been revealed, at the molecular level, to actively contribute to host fitness, particularly nutritional and immunological functions (12, 14). Parasitic nematodes are universally present in the digestive tracts of many animals, and although they were previously considered to only be exploited for host nutritional benefits, mutualistic relationships (such as symbiotic relationships) that have been established over their long evolutionary history are now known to exist (2). Parasitic nematodes and bacteria in the animal gut have co-evolved and established biological interactions, and their balance is considered to be indispensable for host health.

Cockroaches belong to the order Blattodea, 4,641 species have been identified to date, and they preferentially inhabit tropical and temperate forests (4). Parasitic gut nematodes belonging to the family *Thelastomatidae* have been reported in many Blattodea species (1). The long evolutionary history, strong environmental adaptability, and omnivorous traits of cockroaches make them one of the most interesting models for studying co-evolution with parasitic nematodes (24) and the gut microbiome (21). Regardless of the species, cockroach gut communities are modulated by their diet composition, origin (wild versus lab-reared) (5), and developmental stages (adults versus nymphs) (7). There is also evidence that the close evolutionary history between some cockroaches and termites is reflected in the similarities between their gut bacterial communities (27). Although they are generally considered to be non-social species, cockroach gut communities have been suggested to be dominated by bacterial species common in the environment, mostly transmitted through coprophagy (14). The only bacterial symbiont that is vertically transmitted is *Blattabacterium*, which actively participates in nitrogen recycling from stored uric acid (UA) and in the production of essential and non-essential amino acids (7, 26). The elimination of endosymbionts from *Periplaneta americana* by antibiotic treatments resulted in poor growth and a decrease in reproductive capacity (10), thereby emphasizing their importance to the host cockroach.

The combination of species between host cockroaches and parasitic nematodes is mostly fixed. *Leidynema appendiculatum* is mainly found in the hindgut of the smokybrown cockroach *P. fuliginosa*, but is able to artificially infect a broad range of cockroach species (Ozawa, S. *et al.* 2014. Abstract for 6^th^ International Congress of Nematology. p. 215. Cape Town). The American cockroach, *P. americana*, in Japan was found to be co-infected with *Hammerschmidtiella diesingi* and *Thelastoma bulhoesi* (Ozawa, S. *et al.* 2014. Abstract for 6^th^ International Congress of Nematology. p. 215. Cape Town). These thelastomatid nematodes have evolved as general parasites of cockroach species and may be shared between different cockroach species due to the co-existence of their hosts in the same niche (16, 23, 24). Although the existence of these parasitic nematodes in the cockroach gut has already been demonstrated, their role currently remains unknown. Previous studies (9, 19) indicated that parasitized cockroaches were generally longer and heavier, and that different nematode species such as *H. diesingi*, *T. bulhoesi*, and *L. appendiculatum* lacked trophic segregation (*i.e.*, competition), but feeding rates differed (*i.e.*, *L. appendiculatum* consume more than *H. diesingi*). The diets of these nematodes consist of bacterial flora within the host’s gut and the end-products of digestion (9). The effects of these thelastomatid nematodes on the resident hindgut microflora, and, consequently, host fitness currently remain unknown. Therefore, we herein investigated a novel perspective in the insect gut microbiome, namely, the microbial composition of the complete gut (fore-, mid-, and hindgut) microflora of *P. fuliginosa* naturally infected with *L. appendiculatum* and nematode-free, and also analyzed the hindgut microbial communities of *P. americana* naturally co-infected with *H. diesingi* and *T. bulhoesi*, artificially infected with *L. appendiculatum*, and nematode-free.

## Materials and Methods

### Cockroach strains and rearing

*P. fuliginosa* EE strain and *P. americana* NC strain were established and have been maintained in the Hasegawa Laboratory (Chubu University, Japan) since 2013 (23, 24). Both cockroach species were reared in plastic boxes (30×44×32 cm) under the following conditions: food PS-A diet (containing flour, corn, defatted soybean, white fish meal, peanut meal, and rice flour) (Oriental Yeast, Tokyo) and pure water were supplied *ad libitum*, and incubated at room temperature (approximately 25°C). In the present study, the following treatments were used: (1) *P. fuliginosa* EE with the natural parasite *L. appendiculatum* (also referred as naturally infected *P. fuliginosa*); (2) *P. fuliginosa* EE without a parasitic nematode (non-infected *P. fuliginosa* or nematode-free); (3) *P. americana* NC with the natural parasites *H. diesingi* and *T. bulhoesi* (naturally infected *P. americana*); (4) *P. americana* NC with the artificial parasite *L. appendiculatum* (artificially infected *P. americana*); and (5) *P. americana* NC without a parasitic nematode (non-infected *P. americana* or nematode-free).

In order to prepare *P. fuliginosa* EE and *P. americana* NC without parasitic nematodes (established in April-July 2014), several oothecae were collected, surface-cleaned with ETOH 70% (v/v), incubated in a plastic dish until larval hatching (24), and then transferred into plastic boxes and reared under the conditions described above. Individuals of the established nematode-free strains were often dissected to confirm the absence of parasitic nematodes.

In order to prepare *P. americana* NC artificially inoculated with *L. appendiculatum* (established in October 2013), *P. americana* NC without parasitic nematodes were initially established as described above (established in August 2013). *L. appendiculatum* gravid females were collected from *P. fuliginosa* EE. Nematode eggs were collected from *L. appendiculatum* gravid females and kept in cockroach Ringer’s solution until the L2 resting stage was reached (12 d at 25°C). Instars of nematode-free *P. americana* NC were fasted for three d before artificial infection. Approximately ten fasted cockroaches were reared in cylindrical plastic cases (13 cm diameter×22.5 cm height) with 2.0 g of bait mixed with approximately 500 of the L2 resting stage nematode eggs, and were then transferred to the conditions described above.

**Fig. 1:**
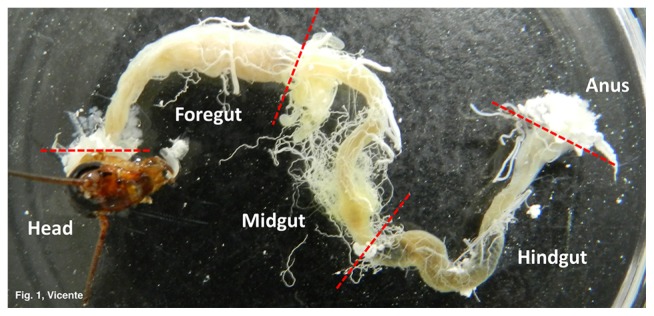
Cockroach gut structure.

Three adult males each of *P. fuliginosa* (average size of treatment 1: 27.2±2.4 mm; and 2: 27.9±1.0 mm) and *P. americana* (average size of treatment 3: 27.9±0.6 mm; 4: 30.0±0.3 mm; and 5: 28.8±0.5 mm) were dissected and the complete alimentary tracts were removed. The tracts were carefully washed in sterile 1× PBS (phosphate-buffered saline), and separated into three sections (fore-, mid-, and hindgut; Fig. 1, Table S1) with sterilized scissors and tweezers. The complete gut of *P. fuliginosa* and only the hindguts of *P. americana* were frozen at −80°C until DNA extraction.

### DNA extraction and checking the presence of nematodes

Frozen gut sections were individually transferred into liquid N_2_ in a sterile mortar and ground with a sterile pestle. Powdered tissue was transferred directly into the lysis buffer (solution C1) of the PowerSoil DNA Isolation kit (MoBio Laboratories, USA), and DNA extraction proceeded accordingly to the manufacturer’s instructions. DNA quality and concentrations were checked using NanoVue plus a spectrophotometer (GE Healthcare Life Science, USA).

The presence/absence of parasitic nematodes in each gut section was confirmed by PCR using specific primers designed from the D2/ D3 region of LSU rRNA 28S (Table S2). PCR was performed in 25-μL reaction mixtures (1× Buffer TAQ polymerase, 0.2 mM dNTP’s, 10 μM Forward/Reverse primer, and 10 ng μL^−1^ DNA) and amplified as follows: denaturation step at 98°C for 30 s; 30 cycles at 98°C for 10 s, 60°C for 30 s, and 72°C for 1 min; and a final extension at 72°C for 5 min. PCR products were purified with NucleoSpin Gel and the PCR clean-up kit (Macherey-Nagel, Germany) and sent for sequencing at Hokkaido System Science (Sapporo, Japan).

### 16S rRNA gene library preparation and pyrosequencing

Intra-variability between individuals was not considered in this study. DNA samples from each gut part (*n*=3) were pooled in equimolar concentrations to fulfil the standard concentration for the 16S rRNA gene library protocol by the Illumina Miseq System. Briefly, 16S V3–V4 regions were amplified using the following conditions: 5 ng μL^–1^ of total DNA, 1 μM of amplicon PCR forward/reverse primers (Table S2), and 2× KAPA HiFi HotStart ReadyMix (KAPA Biosystems) for a 50-μL reaction mixture. PCR amplification was performed with a 3 min denaturing step at 95°C; 25 cycles at 95°C for 30 s, 55°C for 30 s, and 72°C for 30 s; and a final extension at 72°C for 5 min. PCR products were purified with the Agencourt AMPure XP beads kit (Beckman Coulter Genomics). DNA integrity and concentrations were checked with the Qubit dsDNA BR assay kit using the Qubit 3.0 fluorometer (Life Technologies, USA). 16S libraries (*n*=9) were sequenced by Hokkaido System Science using Illumina MiSeq 300 bp Pair-End (301 cycles×2).

### Taxa identification and biodiversity analyses

The post-sequencing processing analysis was conducted in QIIME version 1.9.1 (6). Quality filtering of de-multiplexed reads was conducted using the script *split_libraries.py* with a quality score window (-w) of 50, a maximum number of errors in the barcode (-e) of 0, and considering reverse primer mismatches and truncating at the first N encountered (-z). OTU (Operational taxonomic unit) picking, taxonomy (97% level), and bacterial alignment were conducted using *pick_open_reference_otu.py* with the USEARCH method (13) and 13_8 Greengenes as a reference database. A second level of quality-filtering based on OTU abundance (OTUs lower than 0.005%) was performed as recommended by Navas-Molina *et al.* (22). The presence of *Blattabacterium* in the cockroach gut microbiota is influenced by the dissection procedure, particularly the amounts of residual fat bodies present; thus, this endosymbiont genus was removed from the metadata (28).

In the core diversity analysis, the sampling depth was adjusted for the lowest number of sequences of each library and after rarefaction plot inspection (Fig. S1). The following alpha-diversity estimates were computed: Chao 1 estimator (8), OTUs observed, phylogenetic diversity (PD), and Shannon (H′) and Simpson (1-D) indexes. In order to compare community structures, the taxonomic metric Bray-Curtis (which accounts for the composition and abundance of OTUs) and phylogenetic unweighted UniFrac metrics (which accounts for the composition and phylogenetic distance of OTUs) were used. PCoA (principal coordinates) (18) plots were performed for both distance metrics. In order to infer significant differences (*P*-value with Bonferroni correction less than 0.01) between libraries, *beta_significance.py* was calculated between each pair of libraries and unweighted UniFrac with 999 Monte Carlo permutations were used to test significance.

All libraries were deposited in the NCBI database via SRA (Sequence Read Archive) under the BioProject accession numbers SRP070539 and SRP071767 (Table S3).

## Results

Before pyrosequencing, all samples were checked by PCR for the presence and absence of parasitic nematodes (*L. appendiculatum*, *H. diesingi*, and *T. bulhoesi*) using specific primers designed in the D2/D3 fragment of the 28S rRNA gene (Table S2). The results obtained from the infected nematode *P. fuliginosa* EE and *P. americana* NC clearly showed specific amplification with the predicted size, which was confirmed by Sanger sequencing (data not shown). These results were also supported by the routine dissection of individuals from all treatments in order to confirm the absence of parasitic nematodes.

### Gut microbial composition of *P. fuliginosa* considering natural infection with *L. appendiculatum*

Six 16S rRNA gene (V3–V4 region) libraries were prepared independently (Table S3). The number of sequences obtained ranged between 65,167, and 73,905 after second quality-filtering. In the core diversity analysis (alpha- and beta-diversities), a single rarefaction of 4,528 sequences in each library was performed (Fig. S1A). Table 1 presents the alpha-diversity for each independent library. The observed OTUs (97% level) ranged between 160 in the midgut of non-infected *P. fuliginosa* (L1981) to 549 in the hindgut of infected *P. fuliginosa* (L1978). The observed OTUs (97% level) were higher in *P. fuliginosa* with *L. appendiculatum* than in nematode-free *P. fuliginosa* in all gut sections. The PD index was also higher in *P. fuliginosa* with *L. appendiculatum* (16.99–30.95) than in nematode-free (12.23–15.88) libraries, particularly the hindgut section. The same results were observed for the Shannon (H′) and Simpson (1-D) indexes.

**Fig. 2:**
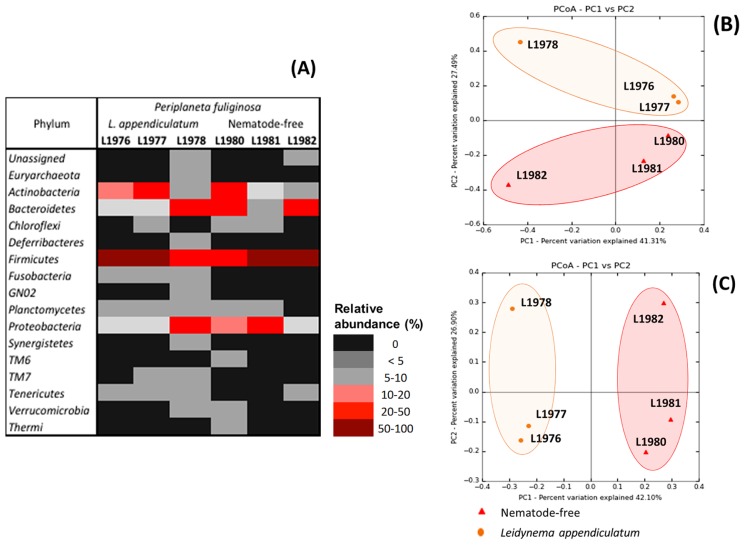
Beta-diversity of the gut community of *Periplaneta fuliginosa* with and without *Leidynema appendiculatum*. (A) Heatmap of phylum relative abundance (%); (B and C) Principal coordinates (PCoA) using Bray-Curtis distance (B) and Unweighted UniFrac. (C).

OTUs were assigned to 16 bacterial phyla: *Euryarchaeota*, *Actinobacteria*, *Bacteroidetes*, *Chloroflexi*, *Deferribacteres*, *Firmicutes*, *Fusobacteria*, GN02, *Planctomycetes*, *Proteobacteria*, *Synergistetes*, TM6, TM7, *Tenericutes*, *Verrucomicrobia*, and *Thermi* (Fig. 1A). The most abundant phylum was *Firmicutes* (Fig. 2A, Table S4) accounting for 73.63%, 50%, and 23.81% in the fore- (L1976), mid- (L1977), and hindgut (L1978), respectively, of infected *P. fuliginosa*, and 30.32%, 69.37%, and 53.34% in fore- (L1980), mid- (L1981), and hindgut (L1982), respectively, of non-infected *P. fuliginosa*. In both treatments, the most abundant genus in *Firmicutes* belonged to the *Bacillales* (*Staphylococcaceae* family) and *Lactobacillales* orders (*Enterococcaeae* and *Lactobacillaceae* families). The *Firmicutes* family’s *Lachnospiraceae* (16.8% in L1981 and 10.8% in L1982) and *Ruminococcaceae* (22.8% in L1982) were also abundant in nematode-free *P. fuliginosa*. In the *Actinobacteria* phylum, the most abundant genera were *Brevibacterium* (4.37% in L1976 and 15.75% in L1977), *Gordonia* (13.94% in L1980), and *Xylanimicrobium* (5.98% in L1977). In the *Bacteroidetes* phylum, *Bacteroides* (26.02%) were the most abundant in the hindgut of non-infected *P. fuliginosa*, followed by the *Weeksellaceae* family (11.97%) in the foregut. *Porphyromonadaceae* (22.4%, L1978) was also detected in infected *P. fuliginosa*. In the case of *Proteobacteria*, *Desulfovibrionaceae* was more abundant in the hindgut of infected *P. fuliginosa* (19.79%) and the order *Enterobacteriales* was found (20.91%) in the midgut of non-infected *P. fuliginosa*.

In terms of beta-diversity, both distances (Bray-Curtis and unweighted UniFrac) reflected the same ordination. In Fig. 2B, L1976–L1977 (fore- and midgut of infected *P. fuliginosa*) clustered closely to L1980–L1981 (fore- and midgut of non-infected *P. fuliginosa*). Unweighted UniFrac was used to assess differences between all pairwise libraries. With the exception of the L1976–L1977 comparison, all communities were considered to be significantly different (*P*-value *Bonferroni*-corrected <0.01) (Table S5).

### Hindgut communities of *P. fuliginosa* and *P. americana* infected with parasitic nematodes

**Fig. 3:**
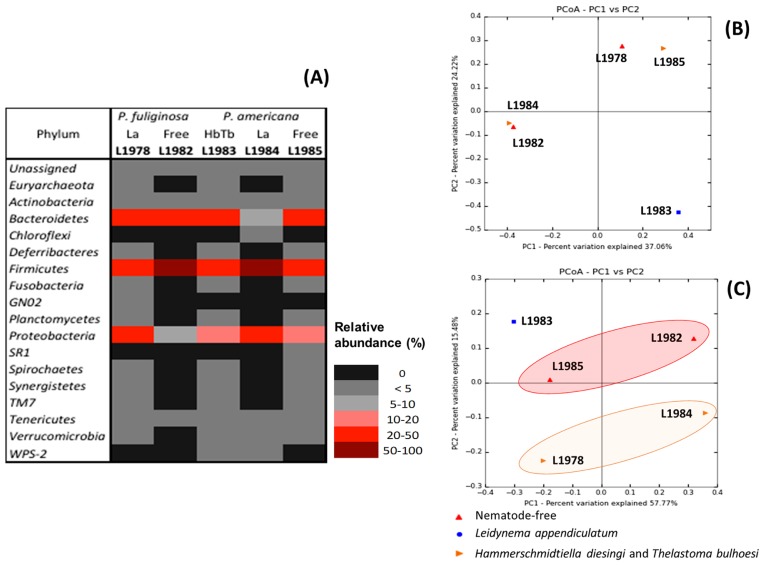
Beta-diversity of hindgut communities of *Periplaneta fuliginosa* and *P. americana* with and without parasitic nematodes (La, *Leidynema appendiculatum*; Hd, *Hammerschmidtiella diesingi*; Tb, *Thelastoma bulhoesi*). (A) Heatmap of phylum relative abundance (%); (B and C) Principal coordinates (PCoA) using Bray-Curtis distance (B) and Unweighted UniFrac. (C).

Five 16S rRNA gene libraries were used for this analysis (Table S3). In the core diversity analysis (alpha- and beta-diversities), a single rarefaction of 5,000 sequences in each library was performed (Fig. S1B). Table 2 presents the alpha-diversity parameters for each library. At the 97% level, OTUs varied between 277 in non-infected *P. fuliginosa* (L1982) to 534 in infected *P. fuliginosa* (L1978). Both cockroaches (*P. fuliginosa* and *P. americana*) had a higher number of OTUs and exhibited higher diversity (PD, H′, 1-D) when infected by their natural parasites. OTUs were assigned to 18 phyla (Fig. 3A, Table S6): *Euryarchaeota*, *Actinobacteria*, *Bacteroidetes*, *Chloroflexi*, *Deferribacteres*, *Firmicutes*, *Fusobacteria*, GN02, *Planctomycetes*, *Proteobacteria*, SR1, *Spirochaetes*, *Synergistetes*, TM7, *Tenericutes*, *Verrucomicrobia*, and WPS-2. The main phyla were: *Bacteroidetes* (11 families), *Firmicutes* (18 families), and *Proteobacteria* (23 families). The more abundant families of *Bacteroidetes* were *Porphyromonadaceae* (20.6%, L1978; 17.5%, L1983) and *Bacteroidaceae* (24.2%, L1982; 12.1% in L1983). In terms of *Firmicutes*, *Ruminococcaceae* (23%, L1982; 34%, L1984; 12.2%, L1985) and *Lachnospiraceae* (11%, L1982) were represented the most. *Desulfovibrionaceae* (19.2%, L1978; 11.6%, L1984) was the only major family in *Proteobacteria*.

The hindgut community of natural-infected *P. americana* (L1983) did not cluster in either ordination analysis (taxonomic, Bray-Curtis; and phylogenetic, unweighted UniFrac) indicating that this community was less similar to the other communities (Fig. 3B and 3C). Considering only the taxonomic relationship (the composition and abundance of OTUs) (Fig. 3B), the hindgut community of non-infected *P. fuliginosa* (L1982) and artificially infected *P. americana* (L1984) clustered together as well as naturally infected *P. fuliginosa* (L1978) and non-infected *P. americana* (L1985). However, when considering their phylogenetic relationship (Fig. 3C), these communities may be grouped into nematodefree (L1982 and L1985) and infected with *L. appendiculatum* (L1978 and L1984). Significant differences (*P*-value *Bonferroni-*corrected< 0.01) between pairwise communities (Table S7) were observed with most of the treatment combinations, with the exception of L1978–L1985 and L1983–L1985.

## Discussion

The gut microbiota of cockroaches is influenced by their origin (wild versus lab-reared) and, consequently, their diet composition (5, 25, 26). In order to avoid this effect, we only worked with lab-reared cockroaches cultured under the same conditions. The major phyla described in lab-reared *P. fuliginosa* (infected with *L. appendiculatum* and nematode-free) and *P. americana* (naturally and artificially infected and nematode-free) were *Firmicutes*, *Bacteroidetes*, and *Proteobacteria*, which have been reported in other cockroach species and suggested to be the normal gut microbiota for conventional cockroaches (20). Pérez-Cobas *et al.* (25) reported that *Bacteroidetes* (*Porphyromonadaceae* and *Rikenellaceae*), *Firmicutes* (*Ruminococcaceae* and *Lachnospiraceae*), and *Proteobacteria* (*Desulfovibrionaceae*) were the main representatives in the gut microbiota of lab-reared and wild *Blattella germanica*. Mikaelyan *et al.* (20) described the same phyla/families for *Shelfordella lateralis*, and Bauer *et al.* (3) for *Panesthia angustipennis* and *Salganea esakii*. *Bacteroidetes* and *Firmicutes* are shared with omnivorous mammals, including humans (7, 11, 20, 28). We found that *Ruminococcaceae*, *Lachnospiraceae*, and *Bacteroidaceae* were more abundant in the hindgut of non-infected *P. fuliginosa*, as well as *Ruminococcaceae* in the hindgut of non-infected *P. americana*. According to previous findings (9), thelastomatid nematodes feed on the end particles of host digestion and also on the bacterial flora of the hindgut. Our results indicate that the hindgut weight of infected cockroaches is higher than that of uninfected cockroaches (Table S1). The size of nematodes ranges, for females, between 2.5 mm for *L. appendiculatum* to 2.8 mm for *H. diesingi*, and 0.7 mm for males (23, 24). Furthermore, the number of nematodes found in the hindgut is high. The *P. fuliginosa* hindgut has been reported to harbor an average of 0.9±0.3 male nematodes and 3.19±2.2 female nematodes (23). In the case of *P. americana*, co-infected by *H. diesingi*/*T. bulhoesi*, the average is 0.7±0.6 male and 1.8±1.6 female *T. bulhoesi* and 1.8±2.1 for male and 1.1±2.0 for female *H. diesingi* (Ozawa, S. *et al.* 2014. Abstracts for 6^th^ International Congress of Nematology. p. 215. Cape Town). In such a small space, the hindgut of parasitized cockroaches appears to be fully compacted, and changes in the resident community may be plausible due to the activity of the nematodes.

Hindgut resident bacteria belonging to *Clostridia* (*e.g.*, *Ruminococcaceae* and *Lachnospiraceae*) and *Bacteroidia* (*e.g.*, *Bacteroidaceae*) are capable of degrading UA, and are considered important in the recycling of UA in termites (30) and cockroaches (10). Even though UA recycling in cockroaches is fundamentally operated by the endosymbiont *Blattabacterium*, we cannot rule out the involvement of these major representatives of the cockroach gut microbiome (26). The *Blattabacterium* genome lacks uricase, which is needed for the initial step of UA degradation (26). Our results showed that the presence of the natural parasitic nematodes altered the bacterial abundance of *Ruminococcaceae*, *Lachnospiraceae*, and *Bacteroidaceae*. Further investigations are needed in order to clarify the role of parasitic nematodes in the nutrient-recycling system of cockroaches.

*Proteobacteria* are more common in wood-feeding termites and other cockroaches (11, 28), and may be related to the composition of the diet. Pérez-Cobas *et al.* (25) found a lower abundance of *Desulfovibrionaceae* in *B. germanica* fed a no-protein regime, suggesting that members of this family, particularly *Desulfovibrio* play a role in nitrogen supply for the host cockroach. Our results demonstrated that naturally infected *P. fuliginosa* and artificially infected *P. americana* had a greater abundance of *Desulfovibrionaceae* than nematode-free *P. fuliginosa* and *P. americana*, even though the diet used was rich in proteins and carbohydrates, thereby supporting the role of parasitic nematodes in nutrient cycling in the host gut.

Previous studies (1, 23) indicate that parasitic nematodes commonly inhabit the hindgut of their host. Nematode eggs are deposited within cockroach feces by gravid females, which are then released by defecation and orally infect new hosts (23). Since the adult stage of these nematodes, in this case *L. appendiculatum*, are mainly found in the hindgut of their hosts, we postulated that their effects in the fore- and midgut microbiota are minimal or absent. Our results showed that although significant differences were observed between the fore- and midgut of infected and non-infected *P. fuliginosa* communities, which were attributed to individual variability, they appeared to be closer phylogenetically than the hindgut of infected and non-infected *P. fuliginosa*. We found that the fore- and midgut of infected and non-infected *P. fuliginosa* had abundant amounts of *Lactobacillales*, particularly the genus *Lactobacillus*. The same findings was reported in lab-reared flies and attributed to diet composition (29).

Mikaelyan *et al.* (20) recently discovered that the natural gut microbiota of *S. lateralis* is able to compete successfully against xenomicrobiota (*i.e.* alien microbial assemblages). The authors established germ-free *S. lateralis* inoculated with foreign gut microbiota, showing that cockroach-specific lineages are more advantageous for the colonization of the gut environment than foreign ones. In the present study, we found that the diversity of the hindgut microbiota of naturally infected *P. fuliginosa* EE and *P. americana* NC were markedly greater than artificially infected or non-infected cockroaches. We hypothesized that natural parasitic nematodes influence the gut microbiome of their hosts, which may have led to shifts in the dynamics of natural bacterial communities. These changes may also be cockroach species-related, although thelastomatid parasitic nematodes may artificially infect a broad range of cockroach species (Ozawa, S., *et al.* 2014. Abstracts for 6^th^ International Congress of Nematology. p. 215. Cape Town).

In the human gut, the presence of helminthes (flatworms and nematodes) has been correlated with alterations in the gut microbiota composition, particularly the excretion/secretion of molecules that change the gut environment (31), which ultimately influences the human immune system (15, 17). The gut microbiota of cockroaches exhibits strong similarities with the human colon (10). We herein for the first time characterized the complete gut microbiota of *P. fuliginosa* and examined the effects of the naturally and artificially infected gut communities of *P. fuliginosa* and *P. americana*. Natural parasites, such as *L. appendiculatum* in *P. fuliginosa* and *H. diesingi*/*T. bulhoesi* in *P. americana*, may have influenced the main representative taxa of these cockroaches. Our results provide basic information and encourage further studies in order to obtain a clearer understanding of the mechanisms and evolution of the host/parasite/microbiota relationship for the establishment of our sustainable society.

## Supplementary Information



## Figures and Tables

**Table 1:** Alpha-diversity of the gut community of *Periplaneta fuliginosa* with and without *Leidynema appendiculatum*

Library		Description		OTU		PD	H′	1-D

				obs	Chao1			
L1976	*Periplaneta fuliginosa*	La	Foregut	232	352	17.01	4.64	0.92
L1977			Midgut	273	394	19.70	5.17	0.94
L1978			Hindgut	549	679	32.21	7.67	0.99
L1980		Free	Foregut	227	245	14.44	5.44	0.93
L1981			Midgut	160	226	12.52	4.65	0.93
L1982			Hindgut	273	338	16.60	6.43	0.97

Species richness (number of OTUs) was estimated with Chao1 (8). Obs and PD indicate the number of OTUs observed in each treatment and phylogenetic diversity, respectively. Diversity and evenness were estimated by Shannon (H′) and Simpson (1-D) indexes.

**Table 2:** Alpha-diversity of hindgut communities of *Periplaneta fuliginosa* and *P. americana* with and without parasitic nematodes (La, *Leidynema appendiculatum*; Hd, *Hammerschmidtiella diesingi*; Tb, *Thelastoma bulhoesi*)

Library		Description		OTU		PD	H′	1-D

				obs	Chao1			
L1978	*Periplaneta fuliginosa*	La	Hindgut	534	667	31.05	7.62	0.99
L1982		Free		277	330	17.02	6.44	0.97
L1983	*Periplaneta americana*	HdTb		445	520	26.92	7.53	0.99
L1984		La		286	315	17.36	6.71	0.98
L1985		Free		398	472	26.38	7.21	0.99

Species richness (number of OTUs) was estimated with Chao1 (8). Obs and PD indicate the number of OTUs observed in each treatment and phylogenetic diversity, respectively. Diversity and evenness were estimated by Shannon (H′) and Simpson (1-D) indexes.
