# Supplementary material for: Composition of the Cockroach Gut Microbiome in the Presence of Parasitic Nematodes
Source: Microbes Environ. 2016 Aug 11;31(3):314–20. doi: 10.1264/jsme2.ME16088 (PMC5017809; doi:10.1264/jsme2.ME16088)
Supplement: Supplementary file 1 [file 31_314_s1.pdf]

Table S1 - Weight of cockroach gut sections with and without parasitic nematodes (La, *Leidynema appendiculatum* ; Free, nematode-free; Hd, *Hammerschmidtiella diesingi* ; Tb, *Thelastoma bulhoesi*).

Host	Nematode	Gut section	Average (mg)	Standard dev.
Periplaneta fuliginosa	La	Foregut	18.93	17.00
		Midgut	12.33	0.80
		Hindgut	37.67	44.45
	Free	Foregut	13.17	5.86
		Midgut	32.47	21.97
		Hindgut	11.13	6.70
Periplaneta americana	Hd and Tb	Hindgut	16.07	6.26
	La		18.53	8.62
	Free		20.75	6.58

Table S2 - List of primers used in this study. Bold region highlighted in 16S V3-V4 region primers indicate the Illumina overhang adapter sequences.

Primers (5'-3')
Leidynema appendiculatum specific primer based in D2D3 region
Forward GCTGGTTGCCAGGCTCTACTAC
Reverse CCGCACCACCGCAGGCCAGCAT
Hammerschmidtiella diesingi specific primer based in D2D3 region
Forward TGGCTGGATGGCTGTACTACTG
Reverse GCATCATCAGACGAGCCAACAC
Thelastoma bulhoesi specific primer based in D2D3 region
Forward GCTCATGTTGCTGACTGCTTCC
Reverse GCACCACCAAACGGGCTAACGC
16S V3-V4 region
Forward **TCGTCGGCAGCGTCAGATGTGTATAAGAGACAG**CCTACGGGNGGCWGCAG
Reverse **GTCTCGTGGGCTCGGAGATGTGTATAAGAGACAG**GACTACHVGGGTATCTAATCC

Table S3 - Characteristics of 16S rRNA gene amplicons from *Periplaneta fuliginosa* and *P. americana* with and without parasitic nematodes (La, *Leidynema appendiculatum*; Hd, *Hammerschmidtiella diesingi*; Tb, *Thelastoma bulhoesi*)

Libraries			Accession	Gut Section	# Raw Pairs	% Trimmed Pairs	% Joined Pairs	% Cleaned Pairs	Mean Length	Pyrotags[1]
L1976	Periplaneta fuliginosa	*Leidynema appendiculatum*	SRR3178367	Foregut	106,610	99.72	71.79	70.47	420	70819
L1977			SRR3180601	Midgut	114,524	99.70	67.72	66.75	421	73905
L1978			SRR3180617	Hindgut	113,967	99.67	68.89	65.83	416	67956
L1980		Free-nematode	SRR3186995	Foregut	112,314	99.63	68.13	66.29	415	68331
L1981			SRR3186996	Midgut	110,814	99.61	68.07	66.44	418	69720
L1982			SRR3186998	Hindgut	109,556	99.57	69.03	65.06	411	65167
L1983	Periplaneta americana	*Hammerschmidtiella diesingi* and *Thelastoma bulhoesi*	SRR3225595	Hindgut	111,834	99.61	63.97	60.28	406	56071
L1984		Leidynema appendiculatum	SRR3225596	Hindgut	115,686	99.67	69.74	66.07	414	59835
L1985		Free-nematode	SRR3225597	Hindgut	113,802	99.64	68.86	64.80	413	69063

[1] Number of pyrotags (sequences) per library after second quality-filtering

Table S4 - Genus abundance (%) for each library. The rarefaction level was 4528 sequences/library. The rarefaction level was 4,528 sequences in each library.

Kingdom	Phylum	Class	Order	Family	Genus	*Periplaneta fuliginosa*					
						Leidynema appendiculattum			Free-nematode		
						L1976	L1977	L1978	L1980	L1981	L1982
Archaea	Euryarchaeota	Methanomicrobia	Methanosarcinales	Methanosarcinaceae	Methanonimicrococcus	0.00%	0.00%	0.07%	0.00%	0.00%	0.00%
Bacteria	Actinobacteria	Acidimicrobiia	Acidimicobiales	Other		0.00%	0.00%	0.00%	0.20%	0.00%	0.00%
		Actinobacteria	Actinomycetales	Other		0.00%	0.00%	0.02%	0.15%	0.00%	0.00%
				Brevibacteriaceae	Brevibacterium	4.37%	15.75%	0.75%	1.50%	1.46%	0.71%
				Cellulomononadaeceae		0.29%	0.20%	0.00%	0.02%	0.02%	0.00%
				Corynebacteriaceae	Corynebacterium	1.46%	1.48%	0.09%	1.63%	0.04%	0.00%
				Dermabacteraceae	Brachybacterium	1.17%	5.10%	0.22%	0.91%	0.07%	0.00%
				Dietziaceae		0.00%	0.00%	0.00%	0.44%	0.27%	0.00%
				Glycomycetaceae	Glycomyces	0.07%	0.29%	0.04%	0.46%	0.09%	0.00%
				Gordoniaceae	Gordonia	3.16%	2.58%	0.29%	13.94%	1.46%	0.04%
				Intrasporangiaceae		0.04%	0.11%	0.02%	0.09%	0.00%	0.00%
				Microbacteriaceae		0.09%	0.11%	0.04%	0.18%	0.09%	0.00%
					Agromyces	0.00%	0.00%	0.00%	0.38%	0.02%	0.04%
					Leucobacter	0.00%	0.02%	0.00%	0.09%	0.02%	0.00%
					Microbacterium	0.04%	0.09%	0.00%	0.07%	0.00%	0.00%
				Micrococcaceae	Arthrobacter	0.00%	0.00%	0.00%	0.29%	0.00%	0.00%
				Mycobacteriaceae	Mycobacterium	0.04%	0.04%	0.00%	5.08%	0.68%	0.07%
				Norcardiaceae	Rhodococcus	0.02%	0.07%	0.00%	0.31%	0.04%	0.00%
				Norcardioidaceae		0.07%	0.66%	0.02%	0.11%	0.02%	0.00%
					Aeromicrobium	0.00%	0.00%	0.00%	0.22%	0.00%	0.00%
				Promicromonosporaceae	Cellulosimicrobium	0.09%	0.07%	0.00%	1.19%	0.02%	0.02%
					Xylanimicrobium	1.63%	5.98%	0.40%	0.42%	0.00%	0.02%
				Pseudonocardiaceae		0.00%	0.15%	0.02%	0.02%	0.07%	0.00%
					Amycolatopsis	0.00%	0.00%	0.00%	0.09%	0.00%	0.00%
					Pseudonocardia	0.02%	0.09%	0.00%	0.24%	0.02%	0.00%
					Saccharopolyspora	0.00%	0.00%	0.00%	0.18%	0.29%	0.07%
				Sanguibacteraceae	Sanguibacter	0.11%	0.51%	0.04%	0.09%	0.02%	0.00%
				Streptomycetaceae	Streptomyces	0.18%	0.20%	0.00%	0.27%	0.02%	0.00%
		Coriobacteriia	Coriobacteriales	Coriobacteriaceae		0.02%	0.13%	0.11%	0.00%	0.04%	0.02%
		Thermoleophilia	Solirubrobacterales	Other		0.00%	0.00%	0.00%	1.04%	0.13%	0.00%
				Patulibacteraceae	Patulibacter	0.00%	0.18%	0.02%	0.02%	0.07%	0.00%
	Bacteriodetes	Bacteroidia	Bacteroidales	Other		0.04%	0.07%	3.00%	0.00%	0.00%	0.00%
				Bacteroidaceae	Bacteroides	0.64%	0.93%	4.39%	0.18%	0.07%	26.02%
				Porphyromonadaceae	Candidatus Azobacteroides	0.27%	0.29%	5.01%	0.00%	0.00%	0.00%
					Dysgonomonas	0.57%	0.66%	7.64%	0.18%	0.00%	4.68%
					Paludibacter	0.09%	0.18%	1.79%	0.00%	0.00%	0.00%
					Parabacteroides	0.46%	0.33%	5.59%	0.00%	0.00%	0.00%
					Tannerella	0.07%	0.04%	2.34%	0.00%	0.00%	0.00%
				Rikenellaceae		0.42%	0.68%	6.49%	0.04%	0.00%	1.21%
					Rikenella	0.02%	0.00%	0.20%	0.00%	0.00%	0.00%
				Odoribacteraceae	Butyricimonas	0.00%	0.00%	0.13%	0.00%	0.00%	0.00%
					Odoribacter	0.04%	0.11%	1.24%	0.04%	0.00%	0.00%
		Cytophagia	Cytophagaceae			0.40%	0.27%	0.09%	0.15%	0.07%	0.00%
		Flavobacteriia	Flavobacteriales	Blattabacteriaceae		0.00%	0.00%	0.02%	1.19%	0.00%	0.00%
		Flavobacteriia	Flavobacteriales	Flavobacteriaceae	Myroides	0.00%	0.00%	0.00%	0.35%	0.00%	0.00%
		Flavobacteriia	Flavobacteriales	Weeksellaceae		0.13%	0.29%	0.00%	11.97%	0.02%	0.00%
		Sphingobacteriia	Sphingobacteriales	Sphingobacteriaceae		0.53%	0.95%	0.15%	3.22%	0.00%	0.00%
		Sphingobacteriia	Sphingobacteriales	Sphingobacteriaceae	Sphingobacterium	0.15%	0.46%	0.09%	1.74%	0.07%	0.02%
		Saprospirae	Saprospirales	Chitinoophagaceae		1.13%	0.42%	0.07%	1.50%	0.00%	0.00%
		Saprospirae	Saprospirales	Chitinoophagaceae	Niabella	0.07%	0.00%	0.00%	0.62%	0.00%	0.00%
	Chloroflexi	Thermomicrobia	AKYG1722			0.00%	0.02%	0.00%	0.42%	0.00%	0.00%
	Chloroflexi	Thermomicrobia	JG30-KF-CM45			0.04%	0.33%	0.00%	2.19%	0.31%	0.02%
	Cyanobacteria	Chloroplast	Streptophyta			0.00%	0.02%	0.00%	0.04%	0.02%	0.00%
	Deferribacteres	Deferribacteres	Deferribacterales	Deferribacteraceae	Mucispirillum	0.00%	0.00%	0.66%	0.00%	0.00%	0.00%
	Firmicutes	Bacilli	Bacillalles	Bacillaceae		0.00%	0.11%	0.00%	1.10%	0.02%	0.00%
	Firmicutes	Bacilli	Bacillalles	Bacillaceae	Bacillus	0.93%	0.04%	0.02%	0.00%	0.00%	0.00%
	Firmicutes	Bacilli	Bacillalles	Paenibacillaceae	Paenibacillus	0.02%	0.00%	0.00%	0.18%	0.00%	0.04%
	Firmicutes	Bacilli	Bacillalles	Planococcaceae		1.17%	0.75%	0.04%	0.31%	0.44%	0.04%
	Firmicutes	Bacilli	Bacillalles	Planococcaceae	Lysinibacillus	0.04%	0.04%	0.00%	0.11%	0.00%	0.00%
	Firmicutes	Bacilli	Bacillalles	Staphylococcaceae	Jeotgalicoccus	0.18%	0.31%	0.00%	0.13%	0.00%	0.04%
	Firmicutes	Bacilli	Bacillalles	Staphylococcaceae	Staphylococcus	12.74%	11.97%	0.46%	2.34%	5.65%	0.97%
	Firmicutes	Bacilli	Lactobacillales	Other		22.13%	12.72%	1.28%	6.65%	9.70%	0.99%
	Firmicutes	Bacilli	Lactobacillales	Enterococcaceae	Enterococcus	1.02%	1.68%	0.84%	1.57%	10.40%	4.00%
	Firmicutes	Bacilli	Lactobacillales	Lactobacillaceae	Other	13.25%	5.98%	1.24%	0.00%	0.00%	0.00%
	Firmicutes	Bacilli	Lactobacillales	Lactobacillaceae	Lactobacillus	19.63%	13.01%	1.06%	15.44%	16.83%	1.90%
	Firmicutes	Bacilli	Lactobacillales	Lactobacillaceae	Pediococcus	0.35%	0.13%	0.00%	0.02%	0.00%	0.00%
	Firmicutes	Clostridia	Clostridiales			0.27%	0.31%	3.03%	0.11%	0.07%	4.42%
	Firmicutes	Clostridia	Clostridiales	Christensenellaceae		0.09%	0.29%	3.25%	0.15%	0.29%	5.30%
	Firmicutes	Clostridia	Clostridiales	Clostridiaceae		0.00%	0.02%	0.15%	0.00%	0.02%	1.26%
	Firmicutes	Clostridia	Clostridiales	Clostridiaceae	Other	0.02%	0.15%	0.00%	0.02%	0.11%	0.00%
	Firmicutes	Clostridia	Clostridiales	Dehalobacteriaceae		0.00%	0.00%	0.00%	0.00%	0.00%	0.11%
	Firmicutes	Clostridia	Clostridiales	Dehalobacteriaceae	Dehalobacterium	0.00%	0.00%	0.04%	0.00%	0.00%	0.11%
	Firmicutes	Clostridia	Clostridiales	Eubacteriaceae	Anaerofustis	0.02%	0.04%	0.00%	0.00%	0.00%	0.00%
	Firmicutes	Clostridia	Clostridiales	Eubacteriaceae	Pseudoramibacter_Eubacterium	0.00%	0.02%	0.00%	0.00%	0.00%	0.00%
	Firmicutes	Clostridia	Clostridiales	Lachnospiraceae	Other	0.02%	0.00%	0.20%	0.04%	0.00%	0.24%
	Firmicutes	Clostridia	Clostridiales	Lachnospiraceae		0.53%	0.38%	2.45%	0.33%	16.74%	10.27%
	Firmicutes	Clostridia	Clostridiales	Lachnospiraceae	Coprococcus	0.02%	0.00%	0.13%	0.00%	0.00%	0.27%
	Firmicutes	Clostridia	Clostridiales	Peptostreptococcaceae		0.07%	0.84%	0.04%	0.75%	1.97%	0.02%
	Firmicutes	Clostridia	Clostridiales	Ruminococcaceae	Other	0.00%	0.00%	0.09%	0.00%	0.00%	0.04%
	Firmicutes	Clostridia	Clostridiales	Ruminococcaceae		0.42%	0.57%	6.12%	0.84%	3.58%	16.50%
	Firmicutes	Clostridia	Clostridiales	Ruminococcaceae	Anaerotruncus	0.00%	0.00%	0.09%	0.00%	0.00%	0.15%
	Firmicutes	Clostridia	Clostridiales	Ruminococcaceae	Oscillospira	0.02%	0.02%	0.57%	0.13%	0.24%	3.98%
	Firmicutes	Clostridia	Clostridiales	Ruminococcaceae	Ruminococcus	0.00%	0.04%	0.40%	0.00%	0.49%	2.12%
	Firmicutes	Clostridia	Clostridiales	Veillonellaceae		0.02%	0.09%	0.64%	0.00%	0.00%	0.00%
	Firmicutes	Clostridia	Clostridiales	Mogibacteriaceae	Other	0.02%	0.00%	0.07%	0.00%	0.00%	0.00%
	Firmicutes	Clostridia	Clostridiales	Mogibacteriaceae		0.04%	0.04%	0.20%	0.00%	0.02%	0.07%
	Firmicutes	Clostridia	Clostridiales	Mogibacteriaceae	Anaerovorax	0.00%	0.02%	0.02%	0.00%	0.00%	0.00%
	Firmicutes	Clostridia	SHA-98			0.00%	0.00%	0.04%	0.02%	0.00%	0.24%
	Firmicutes	Erysipelotrichi	Erysipelotrichales	Erysipelotrichaceae		0.60%	0.40%	1.30%	0.04%	2.80%	3.25%
	Fusobacteria	Fusobacteriia	Fusobacteriales	Fusiobacteriaceae	Other	0.00%	0.00%	0.22%	0.00%	0.00%	0.00%
	Fusobacteria	Fusobacteriia	Fusobacteriales	Fusiobacteriaceae	Fusobacterium	0.42%	0.09%	2.98%	0.00%	0.00%	0.00%
	GNO2	BD1-5				0.02%	0.02%	0.09%	0.00%	0.00%	0.00%
	Planctomycetes	Planctomycetia	Gemmatales	Isosphaeraceae		0.00%	0.00%	0.00%	0.04%	0.00%	0.00%
	Planctomycetes	Planctomycetia	Pirellulales	Pirellulaceae		0.00%	0.02%	0.02%	0.80%	0.04%	0.02%
	Planctomycetes	Planctomycetia	Plantomycetales	Planctomycetaceae	Planctomyces	0.07%	0.09%	0.04%	2.39%	0.11%	0.00%
	Planctomycetes	vadinHA49	PeHg47			0.07%	0.09%	1.81%	0.00%	0.00%	0.00%
	Proteobacteria	Alphaproteobacteria	Caulobacterales	Caulobacteraceae	Other	0.00%	0.00%	0.00%	0.31%	0.00%	0.00%
	Proteobacteria	Alphaproteobacteria	Caulobacterales	Caulobacteraceae	Brevundimonas	0.40%	0.53%	0.11%	0.33%	0.00%	0.00%
	Proteobacteria	Alphaproteobacteria	RF32			0.00%	0.02%	0.82%	0.00%	0.00%	0.00%
	Proteobacteria	Alphaproteobacteria	Rhizobiales			0.18%	0.38%	0.00%	0.11%	0.04%	0.00%
	Proteobacteria	Alphaproteobacteria	Rhizobiales	Bradyrhizobiaceae		0.00%	0.02%	0.00%	0.07%	0.00%	0.00%
	Proteobacteria	Alphaproteobacteria	Rhizobiales	Bruvellaceae		0.04%	0.86%	0.02%	0.00%	0.00%	0.00%
	Proteobacteria	Alphaproteobacteria	Rhizobiales	Brucellaceae	Ochrobactrum	0.22%	2.32%	0.07%	1.63%	0.57%	0.07%
	Proteobacteria	Alphaproteobacteria	Rhizobiales	Hyphomicrobiaceae	Devosia	0.51%	0.51%	0.13%	0.84%	0.09%	0.00%
	Proteobacteria	Alphaproteobacteria	Rhizobiales	Phyllobacteriaceae		0.00%	0.00%	0.00%	0.04%	0.00%	0.00%
	Proteobacteria	Alphaproteobacteria	Rhizobiales	Phyllobacteriaceae		0.09%	0.31%	0.02%	0.82%	0.15%	0.02%
	Proteobacteria	Alphaproteobacteria	Rhizobiales	Rhizobiaceae	Agrobacterium	0.00%	0.00%	0.00%	0.07%	0.13%	0.04%
	Proteobacteria	Alphaproteobacteria	Rhodobacterales	Rhodobacteraceae		0.00%	0.00%	0.00%	0.02%	0.00%	0.04%
	Proteobacteria	Alphaproteobacteria	Rhodobacterales	Rhodobacteraceae	Paracoccus	0.00%	0.00%	0.00%	0.51%	0.02%	0.00%
	Proteobacteria	Alphaproteobacteria	Rickettsiales			0.00%	0.00%	0.02%	0.00%	0.00%	0.00%
	Proteobacteria	Alphaproteobacteria	Rickettsiales	Rickttsiaceae		0.02%	0.00%	0.00%	0.11%	0.00%	0.00%
	Proteobacteria	Alphaproteobacteria	Sphingomonadales	Sphingomonadaceae		0.00%	0.00%	0.00%	0.11%	0.00%	0.00%
	Proteobacteria	Alphaproteobacteria	Sphingomonadales	Sphingomonadaceae		0.00%	0.00%	0.00%	0.11%	0.00%	0.00%
	Proteobacteria	Alphaproteobacteria	Sphingomonadales	Sphingomonadaceae	Novosphingobium	0.09%	0.09%	0.02%	0.38%	0.00%	0.00%
	Proteobacteria	Alphaproteobacteria	Sphingomonadales	Sphingomonadaceae	Sphingopyxis	0.27%	0.18%	0.00%	0.15%	0.00%	0.00%
	Proteobacteria	Betaproteobacteria	Burkholderiales	Alcaligenaceae		0.31%	0.33%	0.02%	2.05%	0.27%	0.02%
	Proteobacteria	Betaproteobacteria	Burkholderiales	Alcaligenaceae	Achromobacter	0.00%	0.04%	0.00%	0.07%	0.02%	0.00%
	Proteobacteria	Betaproteobacteria	Burkholderiales	Alcaligenaceae	Tetrathiobacter	0.02%	0.60%	0.00%	1.08%	0.31%	0.00%
	Proteobacteria	Betaproteobacteria	Burkholderiales	Comamonadaceae		0.02%	0.00%	0.18%	0.00%	0.00%	0.00%
	Proteobacteria	Betaproteobacteria	Burkholderiales	Comamonadaceae	Delftia	0.02%	0.04%	0.00%	0.02%	0.02%	0.00%
	Proteobacteria	Betaproteobacteria	Burkholderiales	Oxalobacteraceae		0.00%	0.00%	0.04%	0.00%	0.00%	0.00%
	Proteobacteria	Betaproteobacteria	Burkholderiales	Oxalobacteraceae	Oxalobacter	0.00%	0.00%	0.02%	0.00%	0.00%	0.00%
	Proteobacteria	Betaproteobacteria	Nitrosomonales	Nitrosomonadaceae		0.00%	0.00%	0.07%	0.00%	0.00%	0.00%
	Proteobacteria	Betaproteobacteria	Nitrosomonales	Nitrosomonadaceae	Nitrosovibrio	0.00%	0.00%	0.00%	0.18%	0.00%	0.00%
	Proteobacteria	Deltaproteobacteria	Desulfarculales	Desulfarculaceae		0.07%	0.07%	2.92%	0.00%	0.00%	0.00%
	Proteobacteria	Deltaproteobacteria	Desulfarculales	Desulfarculaceae	Desulfarculus	0.00%	0.00%	0.02%	0.00%	0.00%	0.00%
	Proteobacteria	Deltaproteobacteria	Desulfobacterales	Desulfarculaceae		0.00%	0.00%	1.21%	0.00%	0.00%	0.00%

	Proteobacteria	Deltaproteobacteria	Desulfobacterales	Desulfarculaceae	Desulfobulbus	0.02%	0.02%	0.97%	0.00%	0.00%	0.00%	
	Proteobacteria	Deltaproteobacteria	Desulfobacterales	Desulfovibrionaceae		0.38%	0.80%	19.79%	0.09%	2.05%	5.65%	
	Proteobacteria	Deltaproteobacteria	Desulfobacterales	Desulfovibrionaceae	Desulfovibrio	0.00%	0.00%	0.02%	0.00%	0.00%	0.00%	
	Proteobacteria	Deltaproteobacteria	Myxococcales			0.00%	0.00%	0.00%	0.15%	0.00%	0.00%	
	Proteobacteria	Deltaproteobacteria	Myxococcales	Haliangiaceae		0.00%	0.00%	0.00%	0.15%	0.00%	0.00%	
	Proteobacteria	Epsilonproteobacteria	Campylobacterales	Campylobacteraceae		0.02%	0.07%	0.07%	0.00%	0.00%	0.00%	
	Proteobacteria	Gammaproteobacteria	Enterobacteriales			0.00%	0.02%	0.00%	0.51%	20.91%	2.54%	
	Proteobacteria	Gammaproteobacteria	Enterobacteriales			0.15%	0.09%	0.02%	0.11%	0.00%	0.00%	
	Proteobacteria	Gammaproteobacteria	Enterobacteriales	Enterobacteriaceae	Enterobacter	0.00%	0.00%	0.00%	0.04%	0.00%	0.07%	
	Proteobacteria	Gammaproteobacteria	Enterobacteriales	Enterobacteriaceae	Serratia	2.58%	0.31%	0.00%	0.27%	0.02%	0.00%	
	Proteobacteria	Gammaproteobacteria	Pseudomonadales	Moraxellaceae	Acinetobacter	1.57%	0.86%	0.07%	0.40%	0.07%	0.00%	
	Proteobacteria	Gammaproteobacteria	Pseudomonadales	Pseudomonadaceae	Pseudomonas	0.46%	0.20%	0.07%	0.62%	0.07%	0.07%	
	Proteobacteria	Gammaproteobacteria	Xanthomonadales	Xanthomonadaceae		0.00%	0.00%	0.09%	0.00%	0.00%	0.00%	
	Proteobacteria	Gammaproteobacteria	Xanthomonadales	Xanthomonadaceae	Luteimonas	0.18%	0.68%	0.09%	0.04%	0.00%	0.00%	
	Proteobacteria	Gammaproteobacteria	Xanthomonadales	Xanthomonadaceae	Stenotrophomonas	0.02%	0.04%	0.04%	0.22%	0.11%	0.04%	
	SR1					0.00%	0.04%	0.02%	0.00%	0.00%	0.00%	
	Spirochaetes	Spirochaetes	Spirochaetales			0.00%	0.00%	0.02%	0.00%	0.00%	0.00%	
	Spirochaetes	Spirochaetes	Spirochaetales			0.00%	0.00%	0.02%	0.00%	0.00%	0.00%	
	Synergistetes	Synergistia	Synergistales			0.00%	0.02%	0.11%	0.00%	0.00%	0.00%	
	TM6	SJA-4				0.00%	0.04%	0.00%	1.02%	0.04%	0.02%	
	TM7	TM7-3	CW040			0.04%	0.00%	0.07%	0.00%	0.00%	0.00%	
	TM7	TM7-3	CW040			0.00%	0.11%	0.29%	0.00%	0.00%	0.00%	
	TM7	TM7-3	I025			0.00%	0.02%	0.00%	0.00%	0.00%	0.00%	
	Tenericutes	CK-1C4-19				0.02%	0.00%	0.00%	0.00%	0.00%	0.00%	
	Tenericutes	Mollicutes	Acholeplasmatales	Acholeplasmataceae		0.02%	0.02%	0.35%	0.00%	0.00%	0.00%	
	Tenericutes	Mollicutes	Acholeplasmatales	Acholeplasmataceae	Acholeplasma	0.00%	0.00%	0.11%	0.00%	0.00%	0.00%	
	Tenericutes	Mollicutes	Anaeroplasmatales	Anaeroplasmataceae	Anaeroplasma	0.00%	0.00%	0.00%	0.00%	0.04%	1.94%	
	Tenericutes	Mollicutes	RsaHF231			0.07%	0.15%	0.00%	0.00%	0.00%	0.00%	0
	Verrucomicrobia	Verrucomicrobiae	Verrucomicrobiales	Verrucomicrobiaceae	Akkermansia	0.00%	0.00%	0.13%	0.00%	0.00%	0.00%	< 5
	Verrucomicrobia	Verrucomicrobiae	Verrucomicrobiales	Verrucomicrobiaceae	Luteibacter	0.00%	0.00%	0.00%	0.07%	0.00%	0.00%	5-10
	WP2					0.00%	0.00%	0.00%	0.02%	0.00%	0.00%	10-20
	Thermi	Deinococci	Deinococcales	Trueperaceae		0.00%	0.00%	0.00%	0.24%	0.00%	0.00%	20-50
Unassigned						0.04%	0.02%	1.90%	0.02%	0.00%	0.15%	50-100

Table S5 - Statistical differences between all pairwise libraries comparisons.

sample 1	sample 2	p value	p value (Bonferroni corrected)
L1976	L1977	0.03	0.45
L1976	L1978	0.00	<=1.0e-02
L1976	L1980	0.00	<=1.0e-02
L1976	L1981	0.00	<=1.0e-02
L1976	L1982	0.00	<=1.0e-02
L1977	L1978	0.00	<=1.0e-02
L1977	L1980	0.00	<=1.0e-02
L1977	L1981	0.00	<=1.0e-02
L1977	L1982	0.00	<=1.0e-02
L1978	L1980	0.00	<=1.0e-02
L1978	L1981	0.00	<=1.0e-02
L1978	L1982	0.00	<=1.0e-02
L1980	L1981	0.00	<=1.0e-02
L1980	L1982	0.00	<=1.0e-02
L1981	L1982	0.00	<=1.0e-02

Unweighted UniFrac distance matrix was used as a significance test with 999 Monte Carlo permutations.

Table S6- Genus abundance (%) for each library. The rarefaction level was 5000 sequences/library. (La, *Leidynema appendiculatum*; Free, Free-nematode; Hd, *Hammershmidtella diesingi*; Tb, *Thelastoma bulhoesi*).

Kingdom	Phylum	Class	Order	Family	Genus	*P. fuliginosa*		*P. americana*		
						La	Free	HdTb	La	Free
						L1978	L1982	L1983	L1984	L1985
Archaea	Euryarchaeota	Methanomicrobia	Methanosarcinales	Methanosarcinaceae	Methanobrevibacter	0.00%	0.00%	0.10%	0.00%	0.00%
					Methanonimicrococcus	0.12%	0.00%	0.34%	0.00%	0.50%
		Thermoplasta	E2	Methanomassiliicoccaceae	vandinC11	0.00%	0.00%	0.10%	0.00%	0.00%
Bacteria	Actinobacteria	Actinobacteria	Actinomycetales	Brevibacteriaceae	Brevibacterium	0.72%	0.60%	0.00%	0.72%	0.06%
				Cellulomononadaeceae	Cellulomonas	0.00%	0.00%	0.00%	0.00%	0.68%
				Corynebacteriaceae	Corynebacterium	0.14%	0.02%	0.00%	0.00%	0.08%
				Dermabacteraceae	Brachybacterium	0.26%	0.00%	0.00%	1.18%	0.04%
				Dietziaceae	Other	0.00%	0.02%	0.00%	0.18%	0.00%
				Glycomycetaceae	Glycomyces	0.02%	0.00%	0.02%	0.18%	0.00%
				Gordoniaceae	Gordonia	0.24%	0.00%	0.02%	0.10%	0.02%
				Intrasporangiaceae		0.02%	0.00%	0.00%	0.02%	0.00%
				Microbacteriaceae	Other	0.02%	0.00%	0.00%	0.10%	0.00%
					Agromyces	0.00%	0.04%	0.00%	0.00%	0.00%
				Micrococcaceae	Other	0.02%	0.00%	0.00%	0.00%	0.04%
					Arthrobacter	0.00%	0.00%	0.00%	0.00%	0.02%
				Mycobacteriaceae	Mycobacterium	0.02%	0.06%	0.00%	0.00%	0.00%
				Norcardioidaceae		0.04%	0.00%	0.00%	0.02%	0.00%
				Promicromonosporaceae	Cellulosimicrobium	0.04%	0.02%	0.00%	0.02%	0.00%
					Xylanimicrobium	0.40%	0.00%	0.02%	0.24%	0.00%
				Pseudonocardiaceae		0.00%	0.00%	0.00%	0.04%	0.00%
					Pseudonocardia	0.06%	0.00%	0.00%	0.02%	0.00%
				Sanguibacteraceae	Sanguibacter	0.00%	0.00%	0.00%	0.48%	0.00%
				Streptomycetaceae	Streptomyces	0.02%	0.02%	0.00%	0.10%	0.00%
		Coriobacteriia	Coriobacteriales	Coriobacteriaceae		0.12%	0.04%	0.74%	0.54%	0.64%
	Bacteriodetes	Bacteroidia	Bacteroidales			3.22%	0.00%	5.36%	0.00%	7.74%
				Bacteroidaceae	Bacteroides	5.46%	24.24%	12.06%	0.00%	4.90%
				GZKB119		0.00%	0.00%	0.14%	0.00%	0.00%
				Porphyromonadaceae	Candidatus Azobacteroides	4.80%	0.00%	1.92%	0.00%	1.80%
					Dysgonomonas	7.46%	5.42%	0.36%	2.22%	1.78%
					Paludibacter	1.46%	0.00%	2.20%	0.00%	0.82%
					Parabacteroides	5.22%	0.00%	12.98%	0.00%	4.60%
					Tannerella	1.70%	0.00%	0.02%	0.00%	0.60%
				Rikenellaceae		5.78%	1.10%	1.80%	0.00%	8.18%
					Rikenella	0.10%	0.00%	0.20%	0.00%	0.30%
				Odoribacteraceae	Butyricimonas	0.06%	0.00%	1.16%	0.00%	1.58%
					Odoribacter	1.78%	0.00%	6.22%	0.00%	1.14%
		Cytophagia	Cytophagales	Cytophagaceae		0.02%	0.00%	0.00%	0.52%	0.00%
					Leadbetterella	0.00%	0.00%	0.00%	0.34%	0.00%
		Flavobacteriia	Flavobacteriales	Flavobacteriaceae		0.00%	0.00%	0.00%	0.08%	0.00%
					Myroides	0.00%	0.04%	0.00%	0.00%	0.06%
				Weeksellaceae		0.04%	0.00%	0.12%	0.00%	0.00%
					Chryseobacterium	0.00%	0.00%	0.00%	0.14%	0.00%
		Sphingobacteriia	Sphingobacteriales	Sphingobacteriaceae		0.06%	0.02%	0.02%	0.32%	0.00%
					Sphingobacterium	0.04%	0.02%	0.04%	0.28%	0.02%
		Saprospirae	Saprospirales	Chitinophagaceae		0.10%	0.00%	0.02%	1.32%	0.00%
	Chroroflexi	Thermomicrobia	JG30-KF-CM45			0.02%	0.02%	0.00%	0.06%	0.00%
	Deferribacteres	Deferribacteres	Deferribacteres	Deferribacteraceae	Mucispirillum	0.64%	0.00%	0.22%	0.00%	1.02%
	Firmicutes	Bacilli	Bacillales	Bacillaceae		0.00%	0.00%	0.00%	0.10%	0.00%
				Planococcaceae		0.10%	0.02%	0.00%	0.00%	0.00%
					Lysinibacillus	0.00%	0.00%	0.00%	0.06%	0.00%
				Staphylococcaceae	Staphylococcus	0.40%	0.92%	0.00%	0.02%	1.84%
			Lactobacillales			1.32%	1.38%	0.28%	2.08%	6.20%
				Enterococcaceae	Enterococcus	0.82%	4.08%	0.52%	5.32%	1.74%
				Lactobacillaceae	Other	1.32%	0.00%	0.00%	0.00%	0.00%
					Lactobacillus	1.14%	2.04%	0.56%	1.16%	2.64%
		Clostridia	Clostridiales			2.70%	4.20%	6.56%	4.48%	3.76%
				Christensenellaceae		3.48%	5.52%	3.24%	5.12%	4.64%
					Chritensenella	0.00%	0.00%	0.00%	0.00%	0.02%
				Clostridiaceae	Other	0.28%	1.06%	0.10%	0.54%	0.04%
						0.02%	0.00%	0.20%	0.00%	0.00%
					Clostridium	0.00%	0.00%	0.04%	0.00%	0.04%
				Dehalobacteriaceae		0.00%	0.04%	0.04%	0.00%	0.00%
				Dehalobacteriaceae	Dehalobacterium	0.10%	0.08%	0.16%	0.10%	0.10%
				Eubacteriaceae	Anarofustis	0.00%	0.00%	0.02%	0.00%	0.02%
				Eubacteriaceae	Pseudoramibacter_Eubacterium	0.00%	0.00%	0.80%	0.00%	0.00%
				Lachnospiraceae	Other	0.36%	0.20%	0.20%	0.54%	0.00%
						2.30%	10.68%	4.20%	7.42%	2.54%
					Anaerostipes	0.04%	0.00%	0.00%	0.80%	0.02%
					Coprococcus	0.22%	0.18%	0.38%	0.46%	0.32%
				Peptostreptococcaceae		0.06%	0.02%	1.06%	0.16%	0.04%
				Ruminococcaceae	Other	0.12%	0.02%	0.00%	0.12%	0.06%
						6.14%	15.82%	8.32%	21.10%	10.04%
					Anaerotruncus	0.14%	0.36%	0.02%	0.26%	0.30%
					Oscillospira	0.72%	4.60%	1.28%	9.38%	1.14%
					Ruminococcus	0.50%	2.16%	0.20%	3.14%	0.66%
				Veillonellaceae		0.88%	0.00%	0.32%	0.00%	0.36%
					Phascolarctobacterium	0.00%	0.00%	0.48%	0.00%	0.00%
				Mogibacteriaceae	Other	0.00%	0.00%	0.64%	0.00%	0.02%
						0.16%	0.10%	0.94%	0.18%	0.18%
					Anaerovorax	0.04%	0.06%	0.26%	0.10%	0.12%
			SHA-98			0.04%	0.30%	1.16%	0.00%	0.02%
		Erysipelotrichi	Erysipelotrichales	Erysipelotrichaceae		1.20%	3.42%	3.88%	1.56%	0.50%
	Fusobacteria	Fusobacteriia	Fusobacteriales	Fusobacteriaceae	Other	0.22%	0.00%	0.06%	0.00%	0.00%
					Fusobacterium	3.14%	0.00%	0.32%	0.00%	0.96%
	GNO2	BD-1				0.14%	0.00%	0.00%	0.00%	0.00%
	Planctomycetes	Planctomycetia	Pirellulales	Pirellulaceae		0.04%	0.00%	0.04%	0.00%	0.46%
			Planctomycetales	Planctomycetaceae		0.00%	0.02%	0.00%	0.02%	0.00%
		vadinHA49	PeHg47			1.84%	0.00%	0.62%	0.00%	1.36%
	Proteobacteria	Alphaproteobacteria	Caulobacterales	Caulobacteraceae	Other	0.00%	0.00%	0.00%	0.02%	0.00%
					Asticcacaulis	0.00%	0.00%	0.00%	1.52%	0.00%
					Brevundimonas	0.10%	0.00%	0.00%	0.06%	0.00%
			RF32			0.86%	0.00%	0.00%	0.00%	0.14%
			Rhizobiales			0.00%	0.00%	0.00%	0.20%	0.02%
				Brucellaceae	Ochrobactrum	0.00%	0.04%	0.00%	0.40%	0.10%
				Hyphomicrobiaceae	Devosia	0.04%	0.00%	0.00%	1.82%	0.00%
				Phyllobacteriaceae		0.04%	0.02%	0.00%	0.10%	0.02%
				Rhizobiaceae	Agrobacterium	0.00%	0.04%	0.00%	0.06%	0.00%
			Rhodobacterales	Rhodobacteraceae	Other	0.00%	0.06%	0.00%	0.00%	0.00%
			Shingomonadales	Sphingomonadaceae	Other	0.02%	0.00%	0.00%	0.02%	0.00%
					Sphingomonas	0.00%	0.00%	0.00%	0.06%	0.00%
					Sphingopyxis	0.02%	0.00%	0.00%	0.02%	0.00%
		Betaproteobacteria	Other			0.00%	0.00%	0.32%	0.00%	0.00%
			Burkholderiales	Alcaligenaceae		0.02%	0.06%	0.00%	0.10%	0.06%
					Achromobacter	0.00%	0.00%	0.00%	0.04%	0.00%
					Tetrathiobacter	0.04%	0.02%	0.00%	0.04%	0.00%
				Comamonadaceae		0.20%	0.00%	0.00%	0.00%	5.76%
				Oxalobacteraceae		0.08%	0.00%	0.08%	0.00%	0.20%
				Oxalobacteraceae	Oxalobacter	0.00%	0.00%	0.04%	0.00%	0.00%
			Nitrosomonadales	Nitrosomonadaceae		0.14%	0.00%	0.00%	0.00%	0.00%
		Deltaproteobacteria	Desulfarculales	Desulfarculaceae		3.32%	0.00%	0.26%	0.00%	1.16%
					Desulfarculus	0.00%	0.00%	0.04%	0.00%	0.04%
				Desulfobacteraceae		1.30%	0.00%	0.86%	0.00%	2.30%
					Desulfosarcina	0.00%	0.00%	0.80%	0.00%	0.00%
					Desulfobulbus	0.86%	0.00%	0.42%	0.00%	0.56%
			Desulfovibrionales	Desulfovibrionaceae		19.14%	6.20%	7.74%	11.58%	6.84%
					Desulfovibrio	0.04%	0.00%	0.00%	0.00%	0.00%
		Epsilonproteobacteria	Campylobacterales	Campylobacteraceae		0.16%	0.00%	0.54%	0.00%	0.00%
					Arcobacter	0.00%	0.00%	3.02%	0.00%	0.00%
		Gammaproteobacteria	Enterobacteriales	Enterobacteriaceae	Other	0.00%	2.62%	0.00%	5.72%	0.26%
						0.00%	0.00%	0.12%	0.22%	0.06%
					Enterobacter	0.00%	0.04%	0.00%	0.00%	0.00%
					Serratia	0.00%	0.00%	0.02%	0.20%	0.00%
			Pseudomonadales	Moraxellaceae	Acinetobacter	0.10%	0.00%	0.00%	0.02%	0.00%
				Pseudomonadaceae		0.00%	0.00%	0.00%	0.00%	0.06%
					Pseudomonas	0.10%	0.02%	0.00%	0.20%	0.00%
			Xanthomonadales	Xanthomonadaceae	Other	0.10%	0.00%	0.00%	0.00%	0.00%
					Luteimonas	0.14%	0.00%	0.02%	0.36%	0.00%
					Pseudoxanthomonas	0.00%	0.00%	0.00%	0.84%	0.00%
					Stenotrophomonas	0.02%	0.04%	0.00%	0.00%	0.00%

	SR1					0.04%	0.00%	0.00%	0.00%	1.42%
	Spirochaetes	Spirochaetes	Spirochaetales	Spirochaetaceae		0.02%	0.00%	0.22%	0.00%	0.06%
					Treponema	0.06%	0.00%	0.02%	0.00%	0.08%
	Synergistetes	Synergistia	Synergistales	Dethiosulfovibrionaceae	TGS	0.10%	0.00%	0.12%	0.00%	0.20%
				Synergistaceae	Candidatus Tammella	0.04%	0.00%	0.00%	0.00%	0.06%
					vadinCA02	0.02%	0.00%	0.46%	0.00%	0.00%
	TM7	TM7-3	CW040			0.02%	0.00%	0.00%	0.00%	0.00%
				F16		0.20%	0.00%	0.12%	0.00%	2.68%
			I025	Other		0.00%	0.00%	0.04%	0.00%	0.00%
	Tenericutes	CK-1C4-19				0.00%	0.00%	0.06%	0.00%	0.00%
		Mollicutes	Acholeplasmatales	Acholeplasmataceae		0.36%	0.00%	0.06%	0.00%	0.00%
					Acholeplasma	0.14%	0.00%	0.12%	0.00%	0.00%
					Anaeroplasma	0.00%	1.74%	0.00%	0.64%	0.00%
			RsaHF231			0.02%	0.00%	0.06%	0.00%	0.06%
	Verrucomicrobia	Opitutae	Opitutales	Opiputaceae	Opitutus	0.00%	0.00%	0.06%	0.00%	0.14%
		Verrucomicrobiae	Verrucomicrobiales	Verrucomicrobiaceae	Akkermansia	0.08%	0.00%	0.00%	0.00%	0.12%
					Luteolibacter	0.00%	0.00%	0.00%	0.18%	0.00%
	WPS-2					0.00%	0.00%	0.10%	0.56%	0.00%
Unassigned						1.86%	0.12%	1.20%	1.54%	0.84%

0
< 5
5-10
10-20
20-50
50-100

The rarefaction level was 5,000 sequences in each library. (La, *Leidynema appendiculatum*; Free, nematode-free; Hd, *Hammerschmidtiella* diesingi; Tb, *Thelastoma bulhoesi*).

Table S7 - Statistical differences between all pairwise libraries comparisons.

sample 1	sample 2	p value	p value (Bonferroni corrected)
L1978	L1982	0.00	<=1.0e-02
L1978	L1983	0.00	<=1.0e-02
L1978	L1984	0.00	<=1.0e-02
L1978	L1985	0.02	0.2
L1982	L1983	0.00	<=1.0e-02
L1982	L1984	0.00	<=1.0e-02
L1982	L1985	0.00	<=1.0e-02
L1983	L1984	0.00	<=1.0e-02
L1983	L1985	0.05	0.5
L1984	L1985	0.00	<=1.0e-02

Unweighted UniFrac distance matrix was used as a significance test with 999 Monte Carlo permutations.

Supplemental Figures legends

Fig. S1. Rarefaction curves based on observed OTUs in each sequence and sample. (A) The rarefaction level was 4,528 sequences in each library, filtering the genus *Blattabacterium*; (B) The rarefaction level was 5,000 sequences in each library, filtering the genus *Blattabacterium.*

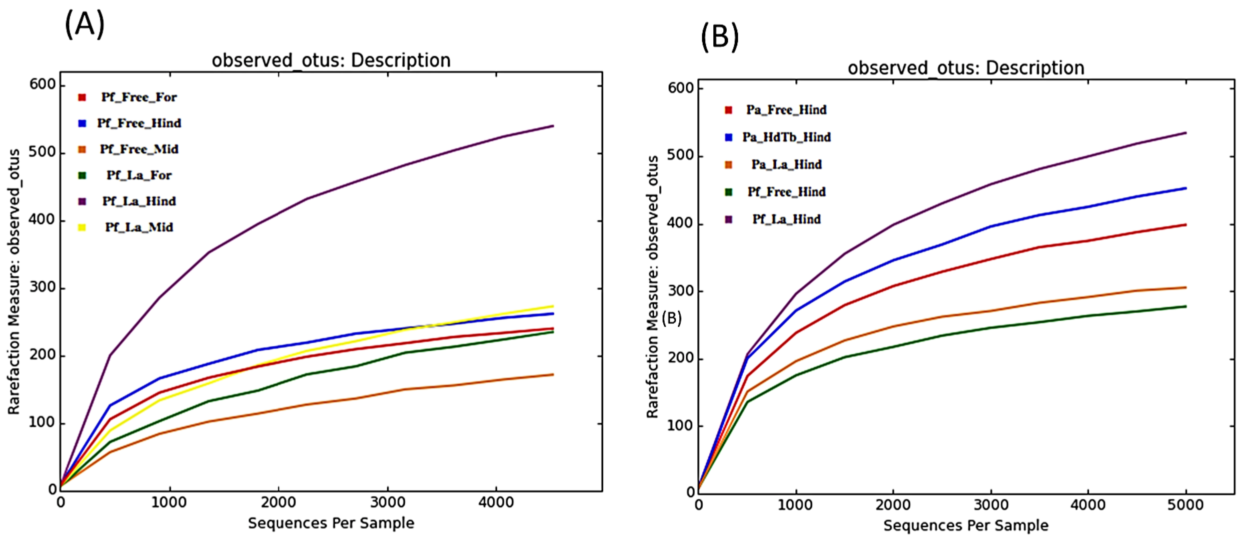

Fig. S1, Vicente
